# Oxygen and Drug-Carrying Periodic Mesoporous Organosilicas for Enhanced Cell Viability under Normoxic and Hypoxic Conditions

**DOI:** 10.3390/ijms23084365

**Published:** 2022-04-14

**Authors:** Ravi Kumar, Nermin Seda Kehr

**Affiliations:** 1 Physikalisches Institute, Westfälische Wilhelms-Universität Münster, Wilhelm-Klemm-Straße 10, 48149 Münster, Germany; ravikapoorsaini@gmail.com; 2 Center for Soft Nanoscience (SON), Westfälische Wilhelms-Universität Münster, Busso-Peus-Straße 10, 48149 Münster, Germany

**Keywords:** nanostructured materials, drug delivery, hybrid materials, oxygen-carrying materials

## Abstract

Over the last decade, inorganic/organic hybrids have been exploited for oxygen-carrying materials and drug delivery. Its low-cost synthesis, controlled shape and size, and stability have made it a viable delivery strategy for therapeutic agents. Rutin (quercetin-3-*O*-rutinoside) is a bioflavonoid found in fruits and vegetables. Rutin has a variety of pharmaceutical applications, but its low water solubility reduces its stability and bioavailability. As a result, we introduce a new and stable nanosystem for loading a low-soluble drug (rutin) into oxygen-carrying periodic mesoporous organosilicas (PMO-PFCs). Over the course of 14 days, this nanosystem provided a sustained oxygen level to the cells in both normoxic and hypoxic conditions. At different pH values, the drug release (rutin) profile is also observed. Furthermore, the rutin-coated PMO-PFCs interacted with both healthy and malignant cells. The healthy cells have better cell viability on the rutin-coated oxygen-carrying PMO-PFCs, while the malignant cells have a lower cell viability.

## 1. Introduction

Inorganic/organic hybrids are the combinations of inorganic and organic building blocks and exhibit their individual properties, which, offer stimulus sensitivity, specific targeting, and controlled delivery of hydrophilic and hydrophobic biomolecules (e.g., drugs) [1,2,3]. Their cost-effective synthesis, controllable shape and pore size, modification by surface chemistry, inertness, and stability have made them an important tool for drug delivery. The large surface area of inorganic nanoparticles (NPs) and uniform organic moieties offers the possibility to adjust the surface hydrophilicity/hydrophobicity and allows fine control of adsorption and release of drug molecules. Researchers have been using various NPs such as mesoporous silica NPs (MSNs), periodic mesoporous organosilicas (PMOs), layered double hydroxides (LDHs), metal NPs (e.g., Au NPs, Ag NPs, etc.), metal oxide NPs (e.g., iron oxide, carbon-based NPs, etc.) for drug delivery [3,4,5,6,7,8,9,10,11]. The sustained and stimulatory drug release can reduce the toxic effects of high-dose drugs in the body. To avoid the burst release of drugs and toxicity of NPs, NPs have been coated with natural or synthetic polymers (e.g., chitosan, alginate, polyethylenimine, polylysine, etc.), lipids/liposomes, cell membranes, dendrimers etc. to improve stability under biological conditions [4,12]. NPs are also suitable for transporting bioactive molecules that are insoluble under physiological conditions, thus improving their bioavailability.

Rutin, also known as rutoside or quercetin-3-*O*-rutinoside, is a naturally occurring bioflavonoid found in vegetables and fruits. Rutin is soluble in organic solvents, e.g., methanol and ethanol, but has poor stability and bio-availability because of its low solubility in water [13,14]. Rutin-coated nanosystems have been demonstrated for various therapeutic objectives over the years and have enhanced aqueous solubility, bioavailability, and stability as well as improved efficiency compared to conventional delivery of rutin [14,15,16,17,18,19,20,21,22]. Different hybrid nanosystems (e.g., zein sodium caseinate nanoparticles [19], gold nanoparticles [23], magnetic nanoparticles [21], carbon nanotubes [24], etc.) have been demonstrated for rutin delivery. However, polymeric and lipid nanoparticles are the most studied nanosystems for rutin [18,25,26,27,28]. Júlio et al. [18] have developed a hybrid nano-polymeric system with rutin and have shown targeted drug delivery for low solubility drugs. Zeng et al. [27] have also designed chitosan-coated mixed phospholipid liposomes (C-CPLs) to increase the biocompatibility of chlorogenic acid and rutin (CA-R) by the oral route. The C-CA-R-CPLs as suitable and novel vehicles were considered for oral targeted delivery and enhanced the oral bioavailability of low-soluble drugs. Kumar et al. [28] developed solid lipid nanoparticles (SLNs) with rutin and demonstrated enhanced oral bioavailability compared to SLNs without rutin and improved bioactivity against hypertension. Researchers have demonstrated numerous rutin-coated nanosystems in vitro, but there are few studies conducted in vivo. Moreover, the bioavailability and stability of such nanosystems have been tested and are well recognized, but the interactions between rutin nanosystems and cells are not widely recognized.

Recently, oxygen (O_2_), the most vital nutrient for cell survival, has been used as a therapeutic agent to improve cell viability and tissue functions under hypoxia conditions. Hypoxic conditions occur when there is insufficient (lower than normal oxygen level) and heterogeneous oxygen distribution within the body tissues, which can reduce cell growth and proliferation. Hypoxia can reduce cell apoptosis, tissue necrosis, transplantation failures, and failures in tissue formation [29]. Over the years, researchers have developed O_2_-releasing biomaterials to provide sufficient and controlled O_2_ delivery to the tissues [30]. Commonly used O_2_ releasing materials are solid inorganic peroxides (e.g., calcium peroxide and sodium percarbonate), hydrogen peroxide, and O_2_ carriers are perfluorocarbons (PFCs) [30,31,32,33,34,35,36,37]. One of the prime examples of hosting O_2_ carrier biomaterials is PMO based nanoparticles [38,39]. Among these studies, the co-administration of O_2_ and drug molecules via NPs and their impact on cell viability have not been sufficiently studied.

Here, in this study, we synthesized the PMO-based nanosystem with oxygen-carrying material (PFC). The internal and external surface of the PMO-PFC system is functionalized with rutin for drug delivery system. Later, the rutin-coated PMO-PFCs are coated with a biodegradable and cell-adhesive bipolymer poly-D-lysine (PDL) for controlled release of rutin. The nanosystem is characterized for zeta potential and size distribution. Further, the nanosystem is measured for its capability to release oxygen under normoxic and hypoxic conditions. The drug release (rutin) profile is also observed at different pH values. To demonstrate the enhanced cell viability under normoxic and hypoxic conditions, the rutin-coated PMO-PFCs interacted with healthy cells and malignant cells. The morphology of the cells is examined by nuclei- and actin-staining. To end this, the PMO-PFC nanosystem shows sustained O_2_ release over a period of 14 days and drug release at different pH values. Rutin and PDL-coated nanosystems show improvement in cell viability for healthy cells under normoxic conditions and a significant decrement in cancer cells under hypoxic conditions.

## 2. Results

### 2.1. Preparation and Characterization of Rutin-Coated Particles

PMO-PFC particles were synthesized according to the previous methods that showed the periodic nature of NPs [40,41,42]. PFCs are chemically inert and hydrophobic organic molecules, and have low solubility in water, but superior O_2_ solubility can exert O_2_ rapidly [43]. Therefore, the internal and external surfaces of the PMO are functionalized with PFCs to avoid the rapid release of O_2_. The hexadecyltrimethylammonium bromide (CTAB) template was removed by adding an acidic solution (HCL) and washing multiple times before drying the final product. These PMO particles have been used in biological studies and show no cytotoxic behavior [10,11,44]. PFC-incorporated PMO particles have shown their ability to provide sustained O_2_ release in hypoxic conditions. Next, rutin was incapsulated into the PMO-PFC system as described in the method section and denoted as Ru(PMO-PFC). Rutin has a wide range of pharmacological properties that have been exploited in human medicine and nutrition. The hydrophobic nature of rutin allows entrapment into PMO particles by hydrophobic forces [45,46], and we observed approximately 92% loading efficiency of rutin into the particles (see calibration curve (Figure S1) and loading efficiency calculation in supporting information). To improve the solubility and stability of the whole system in biological and physiochemical conditions, a biodegradable polymer called “PDL” is used to coat the rutin-coated particles and denoted as Ru(PMO-PFC)PDL. During the PDL coating process, some of the rutin amount is expected to be released into the supernatant. Therefore, we calculated around 83% loading efficiency after the PDL coating into rutin-encapsulated PMO-PFC particles (see supporting information). The functionalized particles were characterized by scanning electron microscopy (SEM), dynamic light scattering (DLS), and zeta potential measurement. The measured zeta potential and average size values are shown in Table 1. The N_2_ sorption experiment and the BET theory were used to determine the porosity and specific surface area of PMO-PFC. The pore size of PMOF was determined by the BJH method (Table S1). The N_2_ sorption isotherm of PMOF shows characteristics for the mesoporous material (Figure S2).

**Figure 1:**
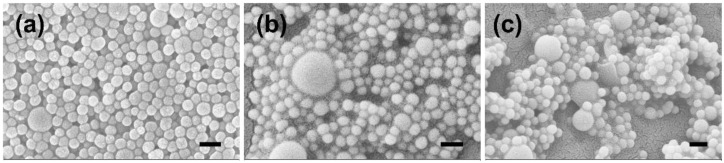
SEM images of PMO-PFC (**a**), Ru(PMO-PFC) (**b**), and Ru(PMO-PFC)PDL (**c**). Scale bars equal 300 nm.

After loading of rutin onto the PMO-PFC particles, the zeta potential changes from −9.93 ± 2.23 to −32.17 ± 1.02 mV, which indicates the successful loading of rutin. This result also indicated the adsorption of rutin on the external surface of PMO-PFC. Further, the size of particles increased from 295 ± 50 to 334 ± 71 nm. After the PDL (positively charged polymer) coating of Ru(PMO-PFC), a positive increase in the zeta potential indicates the successful coating of the polymer matrix on the Ru(PMO-PFC) via electrostatic interaction. The morphology of the nanoparticles is shown in SEM images (Figure 1). The average size of NPs measured by DLS shows a different value than the SEM (143 ± 12 nm). It is because of the aggregation of particles in water due to their hydrophobic nature.

**Figure 2:**
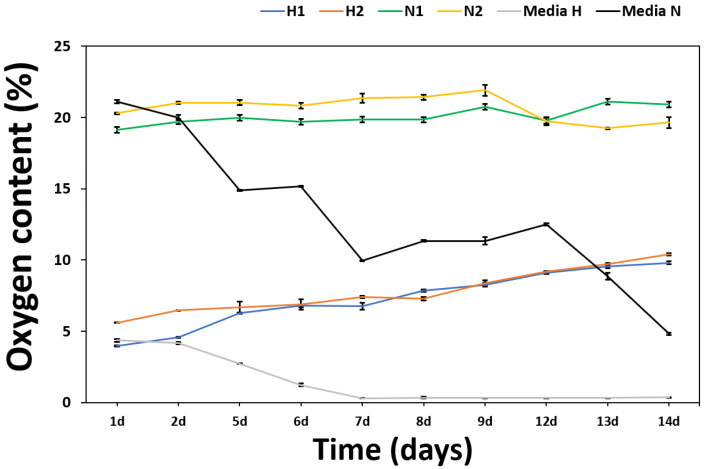
Oxygen content of media containing PMO-PFC particles and particle-free media under normoxic and hypoxic conditions. N1, H1 are the measurement values under normoxic and hypoxic conditions of media with PMO-PFC, respectively. N2, H2 are the measurement values under normoxic and hypoxic conditions, respectively, when PMO-PFC is flushed with 100% O_2_. The number of experiments is three.

To test the oxygen-carrying capacity of PMO-PFC, particles were dispersed in Dulbecco’s modified eagle medium (DMEM) and O_2_ content was measured by an oxygen sensor under normoxic (21% O_2_) and hypoxic (1% O_2_) conditions (Figure 2). First, the media was kept in a hypoxic box at normoxic and hypoxic conditions, then PMO-PFC (1 mg/mL) dispersed in media was added into it (measurements denoted by N1, H1 in Figure 2). In another experiment, first PMO-PFC (1 mg/mL) was dispersed in media flushed with 100% O_2_ in a separate hypoxic box and then added to the media kept in normoxic and hypoxic conditions (measurements denoted by N2, H2 in Figure 2). After adding the 100% O_2_ flushed particles to the media (N2, H2), the O_2_ content normalizes after 1 day for normoxic and hypoxic conditions or reaches the same level as particles without O_2_ flushed (N1, H1) (see Figure S3). Under hypoxic conditions, O_2_ content increases for the PMO-PFC containing media, while particle-free media (Media-H, as control) decreases after 2 days and shows almost zero O_2_ content over the longer period. However, the media with particles in a normoxic condition shows constant O_2_ content over a period of 14 days, while particle-free media (Media-N) continuously decreases. To end this, PMO-PFC particles continuously release oxygen in hypoxic conditions and maintain oxygen levels in normoxic conditions as compared to particle-free media.

### 2.2. Rutin Release Experiment 

**Figure 3:**
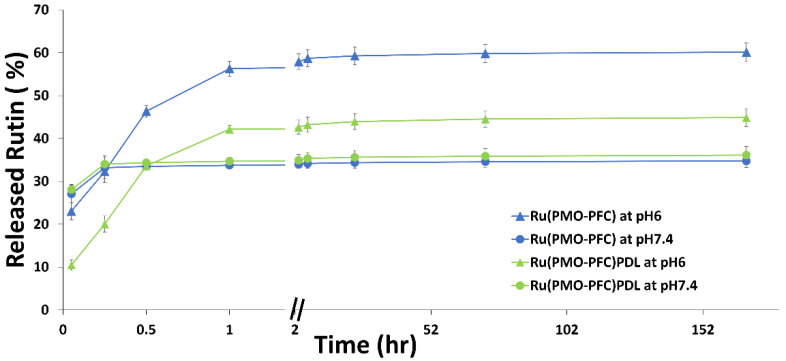
Cumulative percentage rutin release from rutin- and PDL-coated PMO-PFC particles at pH 7.4 and 6.0. Line with triangle markers and round markers are for pH 6.0 and pH 7.4, respectively. The number of experiments is three.

Rutin release from the coated PMO-PFC particles was studied to show the ability of bio-functional particles for controlled and pH-responsive drug delivery. The rutin release was performed at pH 7.4 (the physiological environment) and pH 6.0 (the tumor cell environment). Briefly, the Ru(PMO-PFC) and Ru(PMO-PFC)PDL particles (1 mg/mL) were suspended in the media and stirred for the given incubation time (3 min to 7 days). The absorbance of the collected supernatant after each incubation time was measured by UV spectroscopy, and the concentration of rutin was calculated from the calibration curve. The cumulative percentage release profile (Figure 3) shows release for pH 7.4 and pH 6.0. After 7 d of incubation, 35/60% and 36/45% of rutin were released at pH 7.4/6.0 from Ru(PMO-PFC) and Ru(PMO-PFC)PDL, respectively. It is observed that at pH 6.0, the release in the beginning is slower than the release at pH 7.4. After 1 h, rutin release increased from 23 to 56% for Ru(PMO-PFC) and 10 to 42% for Ru(PMO-PFC)PDL at pH 6.0. However, after 1 h, the rutin released (ca. 1.5–2 µg/mL) is almost constant over the period of 7 days. The higher release at pH 6.0 from rutin-coated particles could benefit anticancer drugs by releasing more drugs into the tumor cell environment. The PDL-coated particles (green line in Figure 3) also show a lower release of rutin because of the biodegradable polymer PDL coating. The PDL coating avoids the sudden release of rutin from the particles. Furthermore, the rutin release profile from Ru(PMO-PFC) and Ru(PMO-PFC)PDL at pH 7.4 and pH 6.0 during a 60 min to 10080 min period was fitted with Higuchi [47] (Figure S4a) and Korsmeyer−Peppas [48] (Figure S4b) models [49]. The rutin release was determined by the *R*-squared value obtained from the kinetic fitting of rutin release. The highest value of the regression coefficient (*R^2^*) proposed a better correlation between the rutin release profile and the kinetic model. The Korsmeyer−Peppas model shows a better *R^2^* value, indicating slow diffusion of the rutin from areas of high concentrations to areas of low concentrations.

To this end, the results show that the amount of rutin released from coated particles depends on the pH of the environment and the coating of the PMO-PFC surface. Therefore, the functionality of PMO-PFC with rutin and PDL is expected to improve bioactivity and control the release of drugs.

### 2.3. Cell Viability Experiment on PMO-PFC, Ru(PMO-PFC), and Ru(PMO-PFC)PDL

**Figure 4:**
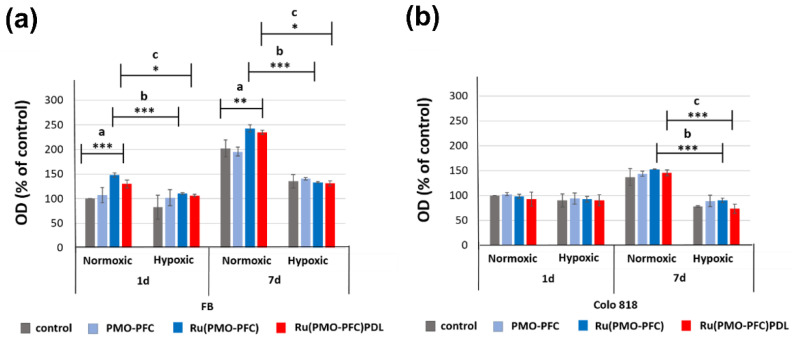
Absorbance (optical density) of the FB (**a**) and colo818 cells (**b**) on a culture plate (control), on a PMO-PFC-coated culture plate, on Ru(PMO-PFC)-coated culture plate, and Ru(PMO-PFC)PDL-coated culture plate under normoxic and hypoxic conditions at 1 and 7 d of incubation. ANOVA: *p* < 0.05 (*), *p* < 0.01 (**), *p* < 0.001 (***); a = significant difference between four groups, b = significant difference between the two groups (between normoxic and hypoxic value of Ru(PMO-PFC)) and c = significant difference between two subgroups (between normoxic and hypoxic value of Ru(PMO-PFC)PDL). The number of experiments is three.

For the sample preparation, particles (1 mg/mL) were first dispersed in ethanol because homogeneous dispersion of PMO-PFCs on the culture plate could not be achieved in DMEM. Then the prepared suspension was then placed into the well of the culture plate. The samples were dried for at least 1 h to make sure the ethanol evaporated. For cell experiments, cells (10^4^) were incubated for 1 d and 7 d on the culture plate at 37 °C with 5% CO_2_. Here, we use primary dermal fibroblasts cells (normal, human, and adult) and human colo818 (malignant melanoma) to see the effect of rutin-coated PMO-PFC particles on healthy and cancer cells, respectively. The cell proliferation reagent WST-1 was used for the spectrophotometric quantification of cell viability. Figure 4 shows the cell experiment data for optical density (OD) versus each sample for the number of incubation days. The data is normalized to the control sample at a 1 d normoxia value for each cell line. The following is a note to the readers: we do not directly compare both cell types as both have different proliferation rates. Here, we demonstrate the impact of particles on different cells under different incubation times and conditions.

We observed that PMO-PFC and rutin-coated PMO-PFC samples (Ru(PMO-PFC) and Ru(PMO-PFC)PDL) show higher optical density (OD) than the control sample (culture plate without particles) at both incubation time under normoxic and hypoxic conditions (Figure 4). However, rutin-coated PMO-PFC samples (Ru(PMO-PFC) and Ru(PMO-PFC)PDL) yield higher viable fibroblast cells (FB) than PMO-PFC for each incubation time. For example, at 1 d of normoxia, we observed ca. 38 and 20% more viable cells for Ru(PMO-PFC)- and Ru(PMO-PFC)PDL-coated samples than for PMO-PFC, respectively (Figure 4a). We expect the ability of PMO-PFC to transport O_2_ and the controlled release of rutin to lead to an increase in cell viability under normoxia and hypoxia. The FB cell viability also increases approximate 82, 63 mand 81% from 1 d to 7 d under normoxia for PMO-PFC-, Ru(PMO-PFC)- and Ru(PMO-PFC)PDL-coated samples respectively (Figure 4a). At 7 d incubation, the cell viability increases 24 and 20% for Ru-(PMO-PFC) and Ru(PMO-PFC)PDL than PMO-PFC. In hypoxic conditions, the cells are deprived of oxygen and this can affect the cell proliferation, but oxygen-carrying particles can release oxygen and increase the proliferation. As results revealed, the cell viability is lower in hypoxia than in normoxia for both incubation times. On the other hand, the FB cell viability under 1 d hypoxia for Ru(PMO-PFC)- and Ru(PMO-PFC)PDL-coated samples increase slightly around 9 and 5% from PMO-PFC, but there are no significant different between these results. For longer incubation time (7 days), we observed an increase in cell viability for all samples. To end this, the rutin-coated and rutin, PDL-coated samples show higher FB viable cells than the PMO-PFC sample under normoxia conditions and for both incubation period; however, these results are not significantly different under hypoxia conditions.

Colo818 cell viability is slightly less for Ru(PMO-PFC) and Ru(PMO-PFC)PDL than PMO-PFC sample at 1 d normoxia (Figure 4b). The Ru(PMO-PFC)PDL-coated sample also shows less viable cell than Ru(PMO-PFC), but no significance difference between group of samples. However, colo818 cell viability increase at longer incubation time under normoxia (approximate 55, 58% more than 1 d normoxia for Ru(PMO-PFC) and Ru(PMO-PFC)PDL respectively). However, under hypoxia conditions at 7 d, the cell viability further decreased, and we observed approximately 2 and 19% fewer viable cells as compared to 1 d hypoxia for Ru(PMO-PFC) and Ru(PMO-PFC)PDL respectively (Figure 4b). The Ru(PMO-PFC)PDL sample also showed 19% fewer viable cells than the PMO-PFC. We understand this could be because of the slow release of rutin from PDL-coated particles. The less viable colo818 cells are because of the added oxygen from the PMO-PFC systems destabilizes the hypoxia-inducible factor (HIFs), which promotes the metastatic potential of tumor cells [26]. Further rutin as anticancer drug kills the cancer cells [50]. Thus, the differences between the viability of FB and Colo818 cells on the PMO-PFC-, Ru(PMO-PFC)-, and Ru(PMO-PFC)PDL-coated culture plate are 36, 32, and 44% at 7 d under hypoxic condition, respectively. To end this, rutin-coated PMO-PFC particles demonstrate enhanced healthy cell viability while simultaneously hindering cancer cell viability over the longer incubation time under hypoxic conditions.

We also performed a control experiment with the same amount of ethanol used to prepare the sample for the cell-viability experiment to investigate the potential toxic effect of the small amount of ethanol (evaporated) on FB cell viability (Figure S5a). Our results showed that the small amount of ethanol added to the sample preparation and evaporated had no significant toxic effects on cell viability. These results were also normalized to the control sample at a 1 d normoxia value for fibroblast cells and compared with the cell viability on a PMO-PFC-coated culture plate (Figure S5b).

### 2.4. Reactive Oxygen Species (ROS)

Oxygen is very important for cell life, but in excess, it is potentially hazardous. ROS is formed when cells exposed to oxygen continuously generate oxygen free radicals. Studies suggest that the mitochondria of tumor cells produce more ROS than those of healthy cells, as many tumors have a higher rate of mitochondrial DNA mutations than normal human tissue [51]. This has been suggested as one of the reasons for the increased ROS production. Therefore, promoting ROS production can induce higher oxidative stress in tumor cells than that in normal cells, resulting in the selective killing of tumor cells without significantly damaging healthy cells. However, increased ROS could mediate various signaling cascades relating to survival, proliferation, resistance to apoptosis and metastases in pre-cancerous cells as well as cancer cells, which may promote cancer initiation and progression. Therefore, the antioxidant drug rutin can balance the amount of ROS production [50,52]. This results in more cancer cell death than healthy cell death because when enough O_2_ is present, HIFs, which promote the drug resistance of tumor and immortal cells, are degraded by the ubiquitin-proteasome pathway.

**Figure 5:**
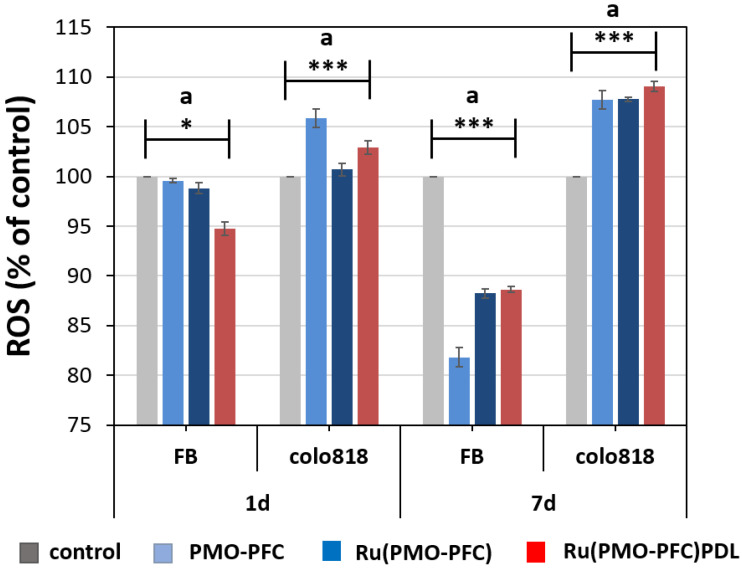
Relative percentages of ROS levels of FB and colo818 cells on the plate, PMO-PFC-, Ru(PMO-PFC)-, and Ru(PMO-PFC)PDL-coated culture plate under hypoxic conditions for 1 d and 7 d. The results were normalized to the control (FB or Colo 818 cells on plate). ANOVA: *p* < 0.05 (*), *p* < 0.001 (***); a = significant difference between four groups. The number of experiments is three.

To investigate the generated ROS, the rutin-coated particles were treated with healthy and cancer cells (Figure 5). First, FB cells and colo818 cells were seeded on the culture plate (control), PMO-PFC-, Ru(PMO-PFC)-, and Ru(PMO-PFC)PDL-coated plate under hypoxic condition. Subsequently, the generated ROS was quantified using 2′,7′–dichlorofluorescin diacetate (DCFH-DA) fluorescent dye. DCFH-DA dye pierced into the cells and ROS oxidized it to fluorescent dichlorofluorescein (DCF). We found that colo818 cells on the plate with PMO-PFC, Ru(PMO-PFC), and Ru(PMO-PFC)PDL particles had a higher ROS level than the control plate. We also observed higher ROS levels for colo818 cells at 7 d than 1 d of incubation time. However, FB cells showed significantly less ROS at 7 d than 1 d of incubation compared with Colo818 cells. A difference of ca. 25% ROS level between FB and colo818 cells at 7 d, suggest that the O_2_ transport capacity of PMO-PFC may enhance the anticancer effect of rutin on cancer cells under hypoxic conditions.

**Figure 6:**
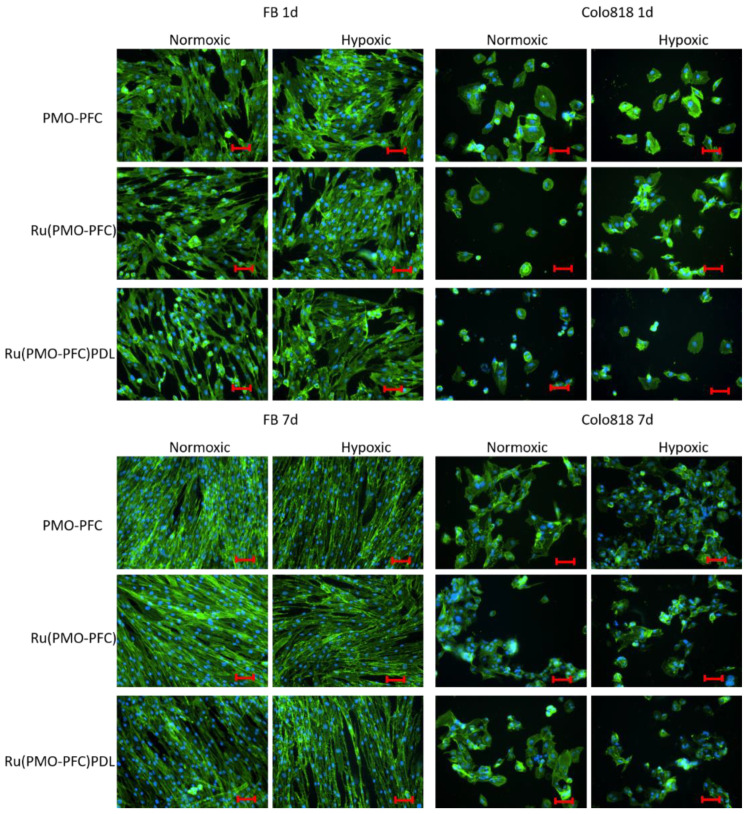
Fluorescence images of FB and colo818 cells on PMO-PFC-, Ru(PMO-PFC)-, and Ru(PMO-PFC)PDL-coated culture plate under normoxic and hypoxic conditions for 1 d and 7 d incubation time. Scale bar equal to 50 µm.

To examine the morphology of the cells on the particle-coated samples, cells were separately seeded on the culture plate. Cells were nuclei stained with Hoechst 33342 dye (blue) and actin-stain with phalloidin Alexa Fluor 488 dye (green). Fluorescence microscopy was used to examine the (Figure 6). We observed that the FB cells in the samples had an elongated and stretched shape, while the colo818 cells were round. It is noted that the number of cells on the images should not be linked with cell viability as some of the cells are washed away during the washing and staining steps.

## 3. Materials and Methods

### 3.1. Materials

Hexadecyltrimethylammonium bromide (CTAB, 98%), [1H,1H,2H,2H perfluorooctyltriethoxysilane (PFC)], [1,2-bis(triethoxysilyl)ethane (BTEE, 96%)], Hoechst 33342 nuclei dye, [2′,7′-dichlorodihydrofluorescein diacetate (DCFHDA)], trypsin, poly-D-lysine (PDL) and the WST-1 assay were ordered from Sigma- Aldrich, Darmstadt, Germany. Rutin-trihydrate was purchased from Roth GmbH, Karlsruhe, Germany. Ammonia (32%), ethanol (absolute, for analysis) and hydrochloric acid (32%, for analysis) were bought from Merck, Darmstadt, Germany. Phalloidin Alexa Fluor 488 was purchased from Invitrogen, Life technology Europe, Bleiswijk, Netherlands. Primary dermal fibroblasts: normal, human, and adult cells were purchased from ATCC, Manassas, VA, USA. Human Colo 818 (malignant melanoma) cells were bought from DSMZ, Braunschweig, Germany. The Dulbecco’s modified eagle medium (DMEM) [supplemented with 1% (*v/v*) penicillin/streptomycin, 2% (*v/v*) L-glutamate, and 10% (*v/v*) fetal bovine serum (FBS)], penicillin/streptomycin, phosphate-buffered saline (PBS), and L-glutamate were obtained from Sigma-Aldrich, Darmstadt, Germany.

### 3.2. Synthesis of Periodic Mesoporous Organosilica (PMO-PFC)

PMO-PFC was synthesized by the modified procedure as follows [41]. Briefly, 30 mL ethanol (99.8%) and 90 mL deionized (DI) water were mixed by magnetic stirrer in a 250 mL round bottom flask. While stirring 485 mg CTAB and 270 μL NH_3_ (32%) were added and stirred for 1 h at room temperature (RT). Then, 1.74 mL (1.67 g, 4.7 m mol) BTEE and PFC (0.59 g, 1.16 m mol, 0.44 mL in 3 mL ethanol) stir for additional 48 h at RT. After 48 h and still stirring 50 mL ethanol (99%) was added, then 1.4 mL HCL (32 wt%) was slowly added to the mixture and stirred for 6 h at 50 °C to remove the CTAB [41]. The reaction mixture was transfer to 15 mL falcon tubes and centrifuged (6500 rpm) for 15 min. The supernatant was discarded, and PMO-PFC particles were washed further 3 times with ethanol by centrifugation. The tubes with particles were left with open lid to dry for 2 days at RT.

### 3.3. Loading of Rutin to PMO-PFC Particles

An amount of 100 mg of PMO-PFC particles and rutin trihydrate (80 mg) were mixed together with 0.4 mL ethanol, sonicated for 20 min, and stirred for 10 min at RT. Then, 1.1 mL DI water was added and sonicated again for 5 min. The mixture was stirred for overnight at RT and then, centrifuged for 15 min to collect the rutin-coated PMO-PFC particles. The coated particles were further washed with DI water and dried overnight at RT. The supernatant was collected for the purpose of measuring rutin concentration. The amount of rutin loaded in the PMO-PFC particles was determined by means of spectrophotometric analyses. A total of 300 μL of supernatant was added to a 96 well plate and the absorbance was measured at 352 nm by a UV-vis spectrometer. The concentration of free rutin was calculated by using a calibration curve. The amount of loaded rutin was the amount of rutin added initially minus the amount of free rutin in the supernatant. The loaded efficiency (E%) was calculated according to the following equation below:

E% = (amount of rutin added-amount of free rutin)/(amount of rutin added) × 100

### 3.4. Coating of Poly-D-Lysine (PDL) to Ru(PMO-PFC) Particles

An amount of 50 mg of Ru(PMO-PFC) was added to 1.5 mL PDL solution (0.5 mg/mL in DI water) in a 2 mL Eppendorf tube and sonicated for 20 min and then stirred for 1 day at RT. After, the mixture was centrifuged for 15 min and the supernatant was used to calculate the released rutin by means of spectrophotometer analysis. The absorbance and E% were measured as explained in above section and supporting information. The product was dried with the open lid at RT.

### 3.5. Rutin Release in DMEM Media

First, 1 mg/mL rutin-loaded particles were suspended in DMEM (pH-6.0 and 7.4) by sonication and stirred in a 2 mL Eppendorf tube. The suspension was centrifuged after each incubation time (3 min to 7 days). The supernatant was transferred into new Eppendorf tube and fresh media was added to the particles and stirred again for next incubation time (adding up the previous incubation time). The collected supernatants were used to measure the absorbance of rutin for each incubation time by spectrophotometric analysis. The concentration of released rutin was calculated by using a calibration curve.

### 3.6. Measurement of O_2_ Content from PMO-PFC

The O_2_ content of cell culture media and PMO-PFC containing cell culture media was determined using an oxygen sensor (OXY-1 SMA trace, Pre Sens Precision Sensing GmbH, Regensburg, Germany). All samples were incubated either in normoxic (21% O_2_) or hypoxic conditions (1% O_2_) for 14 days at RT. To generate the hypoxic condition, we used a hypoxia box saturated with a gas mixture of 1% O_2_ + 5% CO_2_ + 94% N_2_. First, the 500 µL media was placed in a hypoxia box closed with rubber cap and saturated with 1% O_2_, then 500 µL of PMO-PFC (1 mg/mL in media) was added by syringe and oxygen level measured (denoted by H1). In another case, 500 µL of PMO-PFC (1 mg/mL in media) was flushed by 100% O_2_ and then added to 500 µL media in hypoxic box (measurement denoted by H2). Similar experiments were performed for normoxic conditions and measurements are denoted by N1 and N2, respectively.

### 3.7. Cell Viability of Samples on Cell Culture Plates

First, PMO-PFC, Ru(PMO-PFC), and Ru(PMO-PFC)PDL particles (1 mg/mL) were dispersed in ethanol and sonicated for 5 min. Then, the suspensions (20 µL) were placed on a culture plate and dried at RT. The cells were thawed from −80 °C and carefully seeded on the culture plate with pre-warmed media. After overnight incubation at 37 °C and 5% CO_2_, cells were washed with PBS and harvest by trypsinization. Then, cells were collected by adding pre-warmed media and centrifuged for 3 min. The supernatant was discarded, and cell pallet was collected by adding 1 mL pre-warm media. Cells were counted in hemocytometer and the particles-coated plate was incubated with 10^4^ cells for 1 d and 7 d under hypoxic and normoxic condition at 37 °C and 5% CO_2_. For control, the cells were seeded on a plate without particles. For the control experiment, to show the no toxic impact of the small volume of ethanol added during the sample preparation on the FB cell viability, the same amount of ethanol (20 µL) was placed on a culture plate and dried at RT and then used for control cell experiments. After incubation period, the samples with cells were washed with PBS (2×) and incubated with cell proliferation reagent WST-1 assay (10 *vol*% in media) for 4 h. Cell viability was measured at 460 nm by scanning the plate with a UV-vis spectrophotometer.

### 3.8. Co-Staining of Cells

Cells were seeded separately on the samples and incubated for 1 d and 7 d at 37 °C and 5% CO_2_ under hypoxic and normoxic condition. Then, the cells were fixed with paraformaldehyde (4%). After 10 min, samples were washed with PBS (2×) and the nuclei-staining was performed with Hoechst 33342 dye [stock solution (16.2 mM), diluted 1:2000 in PBS] at RT for 10 min. Then, samples were washed with PBS (2×) and incubated with 0.1% Triton X-100 in PBS for 10 min at RT. Samples were then washed with PBS (3×), and co-stained with phalloidin Alexa Fluor 488 [5 µL of the methanolic stock solution (6.6 µM) of phalloidin Alexa Fluor 488, diluted in 200 µL of PBS containing 3% bovine serum albumin] for f-actin staining. Samples were kept overnight at RT and stored in the dark. Afterward, the samples were washed with PBS (2×) and ready for imaging.

### 3.9. Quantification of the Reactive Oxygen Species (ROS)

The cells were seeded separately onto the samples in a 96-well cell culture plate and incubated for 1 d and 7 d at 37 °C under hypoxic (1% O_2_) condition. After the incubation period, all samples were washed with PBS (2×) and then reacted with 2′,7′–dichlorofluorescin diacetate (DCFH-DA, 10 μM) for 30 min at 37 °C in cell culture media. The relative fluorescence intensity was measured (Ex = 485 nm and Em = 530 nm) by a UV-vis spectrophotometer.

### 3.10. Characterization

Scanning electron microscopy (SEM) was performed on a Zeiss cross beam 340 scanning electron microscope to determine the morphology of the particles. The average size of NPs from the SEM image was measured by ImageJ software. Zeta potential measurements and dynamic light scattering (DLS) were conducted using a Malvern Zetasizer Nano Series. A Nikon ECLIPSE Ts2R fluorescence microscope was used to determine the cell morphology and fluorescence imaging. Cell viability was measured using a Tecan Infinite 200 PRO spectrophotometer.

### 3.11. Statistical Methods 

All experiments were performed at least three times. The results are shown as average values with standard deviations. Significance tests were conducted using a single factor ANOVA test. Significance levels were depicted as * for *p* ≤ 0.05, ** for *p* ≤ 0.01, and *** for *p* ≤ 0.001, where *p* is the probability value and a statistical measurement used to validate a hypothesis against observed data.

## 4. Conclusions

We demonstrate a simple yet effective method to encapsulate non-aqueous drug loading and oxygen-carrying periodic mesoporous organosilica PMO-PFC nanomaterial. The loading efficiency of the aqueous drug rutin was estimated at around 92%. Under hypoxic and normoxic conditions, this nanosystem provided O_2_ content for 14 days. The release experiment also suggests that drug release is pH sensitive. The viability of healthy cells was improved by rutin-coated particles in normoxic conditions, according to cell viability tests. Furthermore, the anticancer impact of rutin and O_2_ carrying PMO-PFC particles is supported by the reduction in cell viability of Colo 818 cells under hypoxic conditions. Under hypoxia, rutin-coated PMO-PFC particles resulted in higher cell proliferation of healthy fibroblast cells than malignant colo818 cells. The ROS experiment also revealed that rutin encapsulated O_2_ carrying nanoparticles induced more ROS in colo818 cells. Therefore, the overall performance of the organic-inorganic hybrid PMO-PFC nanosystem makes it an effective approach for loading low-soluble therapeutic agents and improving cell viability. This nanosystem, we anticipate, may be integrated into hydrogels and even used as an injectable O_2_-carrying biomaterial for local drug delivery.

## Figures and Tables

**Table 1:** Zeta potential and size measurement (mean value ± standard deviation) of particles.

Sample	Zeta Potential (mV)	Size (nm)
PMO-PFC	−9.93 ± 2.23	295 ± 50
Ru(PMO-PFC)	−32.17 ± 1.02	334 ± 71
Ru(PMO-PFC)PDL	23.30 ± 0.79	485 ± 67

## Data Availability

Not applicable.

## Supplementary Materials

The following supporting information can be downloaded at: https://www.mdpi.com/article/10.3390/ijms23084365/s1.
