# Supplementary material for: Oxygen and Drug-Carrying Periodic Mesoporous Organosilicas for Enhanced Cell Viability under Normoxic and Hypoxic Conditions
Source: Int J Mol Sci. 2022 Apr 14;23(8):4365. doi: 10.3390/ijms23084365 (PMC9024945; doi:10.3390/ijms23084365)
Supplement: Supplementary file 1 [file ijms-23-04365-s001.zip › ijms-1675445-supplementary.pdf]

Supplementary Information

Oxygen and Drug-carrying Periodic Mesoporous Organosilicas for Enhanced Cell Viability under Normoxic and Hypoxic Conditions

Ravi Kumar [1,2], Nermin Seda Kehr [1,2*]

Dr. Ravi Kumar, Dr. Nermin Seda Kehr

[1] Physikalisches Institute, Westfälische Wilhelms-Universität Münster, Wilhelm-Klemm-Straße 10,

48149 Münster, Germany

[2] Center for Soft Nanoscience (SON), Westfälische Wilhelms-Universität Münster, Busso-Peus-Strasse 10, Münster 48149, Germany; ravikapoorsaini@gmail.com

[*] Correspondence: seda@uni.muenster.de

Loaded efficiency (E%) of rutin into PMO-PFC particles:

E% = (amount of rutin added-amount of free rutin) / (amount of rutin added) × 100 .

From calibration curve (Figure S1 A), Y = 0.01294 x − 0.00276

Absorbance of supernatant after loading of rutin into PMO-PFC (Figure S1 B), Y = 0.7976

Free rutin in supernatant in 1ml, x = 61.85 ~ 62 µg,

Amount of rutin added initially (80 mg) per 100 mg PMO-PFC, means 0.8 mg (800 µg) per 1 mg PMO-PFC

Therefore E% = (800-62)/(800) × 100 = 92.25% ~ 92%

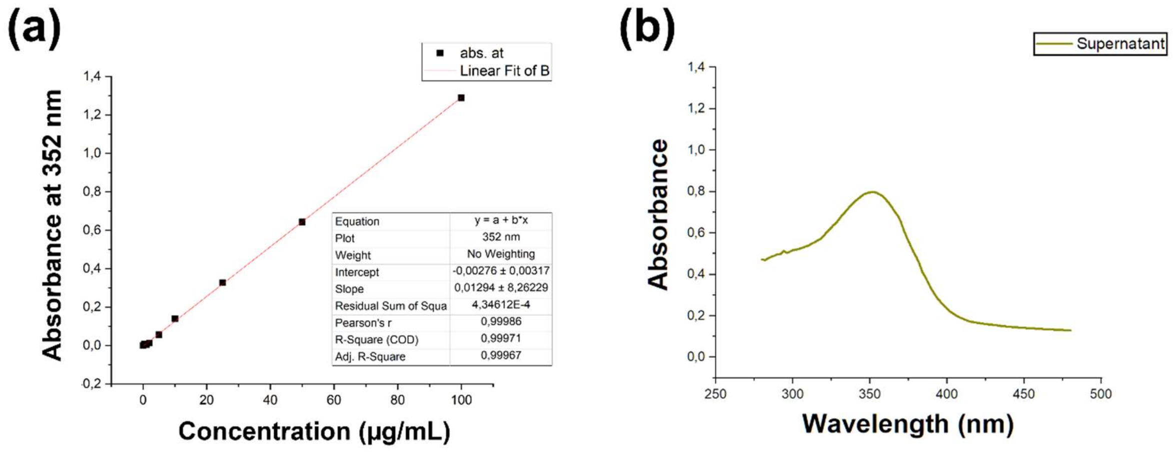

Figure S1. Calibration curve of rutin in water at wavelength of 352 nm (a). The absorbance spectra of supernatant after rutin coating of PMO-PFCs (b).

Similar way after PDL coating, the absorbance of supernatant was measured (Y = 0,8659) and rutin concentration was measured 67 µg/ml. therefore E % = (738-67/ 800)× 100 = 83%.

Table S1. Structural parameters of PMOF.

BET surface area ($m^2\ g^{-1}$)	Pore volume ($cm^3\ g^{-1}$)	Pore size (nm)
449.695	0.269	2.39

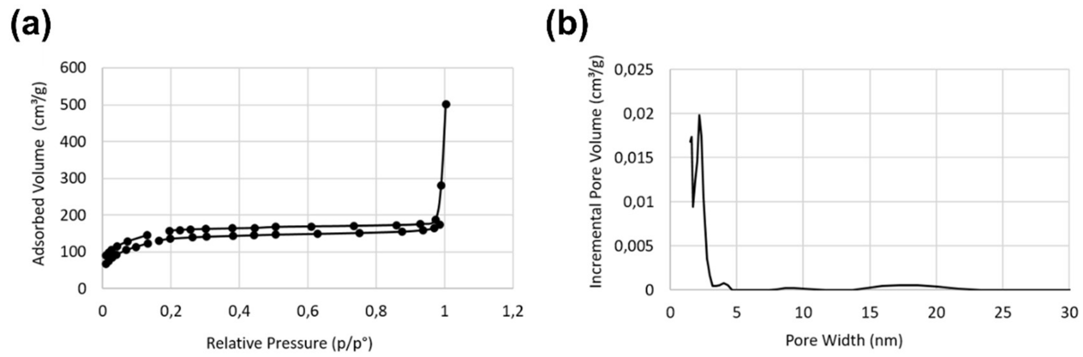

Figure S2. N2 adsorption-desorption isotherm (a) and pore dimeter (b) for PMO-PFC.

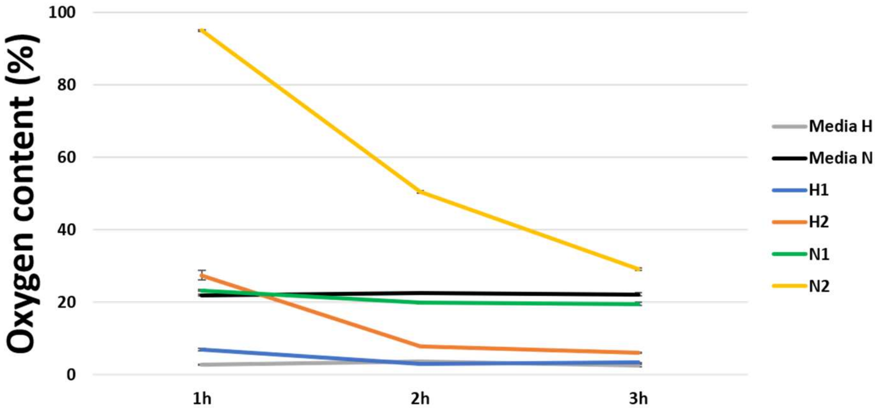

Figure S3. Oxygen content of media containing PMO-PFC particles and particle-free media under normoxic and hypoxic condition. N1, H1 are the measurement value under normoxic and hypoxic condition of media with PMO-PFC, respectively. N2, H2 are the measurement value under normoxic and hypoxic condition respectively, when PMO-PFC flushed with 100% O_2. Number of experiments are 3.

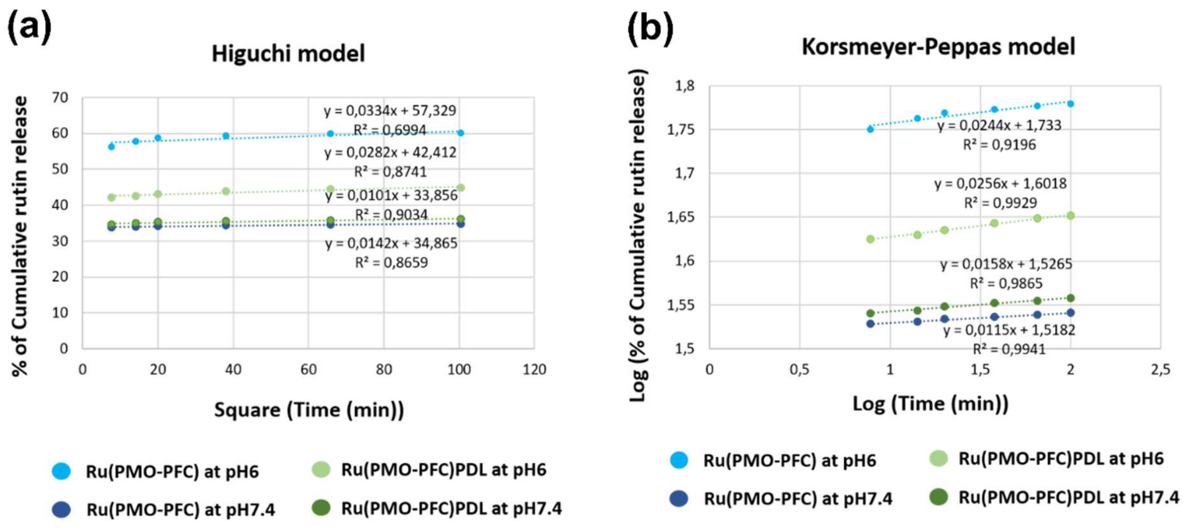

Figure S4. Rutin release profile from Ru(PMO-PFC) and Ru(PMO-PFC)PDL at pH 7.4 and pH 6.0 was fitted with Higuchi (a) and Korsmeyer−Peppas (b) models.

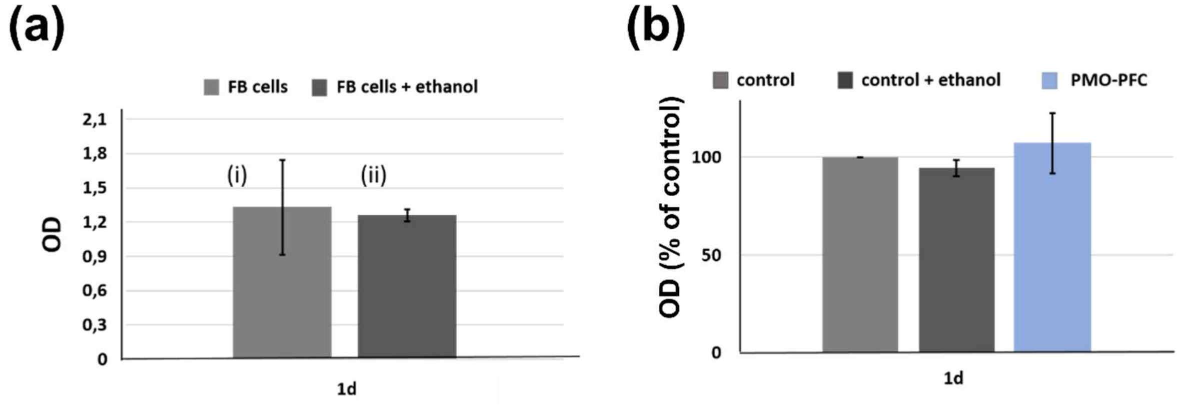

Figure S5. (a) OD of the FB (i) on a culture plate and (ii) on a culture plate treated with the same amount of ethanol used for the preparation of particles on cell culture plate. (b) OD (% of control) of the FB on a culture plate, on a on a culture plate treated with the same amount of ethanol used for the preparation of particles on cell culture plate, and on a PMO-PFC coated culture plate. ANOVA test showed no significant differences between the tested groups. Number of repeated experiments (N) = 3.
